# Marketing of Slow-Track Rather than Fast-Track Animals Reduces Mean Length of Stay and Animal Shelter Census Count: A Theoretical Study

**DOI:** 10.3390/ani16081158

**Published:** 2026-04-10

**Authors:** Michael Loizos Mavrovouniotis

**Affiliations:** SCIL and Social Compassion, P.O. Box 1125, Laguna Beach, CA 92652-1125, USA; mlmavro@yahoo.com

**Keywords:** animal sheltering, length of stay, shelter capacity, capacity for care, animal adoption

## Abstract

Animal shelters operate under capacity constraints in which average length of stay (LOS) determines the number of animals in care and the resources required to maintain humane standards. Shelters commonly distinguish between fast-track animals, which are expected to be adopted quickly, and slow-track animals, which tend to remain longer. Many operational interventions, such as preferential kennel placement or active marketing, are limited resources that must be allocated among these groups. We developed a mathematical framework to evaluate how simple, broadly applicable interventions affect expected LOS as a function of an animal’s baseline LOS. We analyzed the impact of marketing interventions with multiplicatively higher adoption odds. We showed analytically that marketing interventions yield larger reductions in expected LOS when applied to slow-track animals. To examine population-level consequences, we constructed a steady-state kennel allocation example and validate the results using Monte Carlo simulation with stochastic intakes and adoptions. Across both analyses, prioritizing slow-track animals for marketing resources minimizes the total in-care census count relative to the opposite strategy. These findings provide a quantitative basis for allocating limited shelter resources. Preferential marketing of slow-track animals helps reduce mean LOS and maintain the in-care census count within capacity.

## Simple Summary

In animal shelter population management, adoption-promoting resources are limited. A shelter deploys them to reduce the future census count of animals in care. This means that resources should promote the largest possible reduction in length of stay of the animals for which they are used. We assume that marketing interventions such as higher visibility kennels or online promotions of individual animals have a proportional effect on the odds of adoption per day. Under this assumption, we show that marketing interventions are more beneficial when the initial expected length of stay is longer. When a shelter classifies animals as fast- or slow-track, marketing that is equally applicable to all animals should be allocated to slow-track animals first. We get the same result when we include the random arrival and departure of animals in the simulation.

## 1. Introduction

The average length of stay (LOS) across all animals handled by a shelter determines the average number of animals in care, a key metric for the humane and efficient operation of the shelter [1,2]. The number of animals housed has a direct impact on resource requirements, animal health and well-being, and shelter performance measures such as adoption rates [1,2,3]. Shelters have limited capacity for care and must ensure that they remain within that capacity [3,4]. Measures such as accelerating health examinations and vaccinations, holding adoption events, and various forms of marketing can contribute to maintaining the in-care census count within shelter capacity [4].

The optimal use of shelter capacity is increasingly recognized as a multi-dimensional challenge requiring both empirical predictive modeling and theoretical frameworks. Recent scholarship is expanding from animal-level predictions to strategic resource allocation, treating time and space as finite resources that must be managed to maintain and improve performance. In a two-phase approach, the animal-level prediction of LOS may guide animal allocation across shelters to improve outcomes and reduce mean LOS [5]. Another systemic approach relies on sensitivity analysis of various performance metrics, to analyze the relative impact of investments in capacity and adoption-related activities [6]. To minimize euthanasia stemming from capacity limitations, efficient sharing of space may be determined via mathematical optimization across shelters [7]. These studies underscore that LOS and census statistics are not merely a side-effect of animal characteristics. Rather, they can be managed and optimized with the available resources.

Building on predictive foundations, a recently proposed framework takes the next step by examining how decisions on individual animals can be rigorously guided by treating time as a strategic resource rather than a uniform constraint [8]. This enables optimal use of limited shelter resources at the population level. This observation aligns with moving animals through the system as efficiently as possible, in order to maintain the census count within the shelter’s capacity for care [9].

In identifying the best ways to get individual animals to their optimal outcome as quickly as possible, it is useful to distinguish between “fast”- and “slow”-track animals [1]. Expected LOS and classification into fast- and slow-track depend on factors such as animal size and age [10,11] and may vary from shelter to shelter [5]. For fast-track animals that appeal to many adopters and are expected to leave quickly, shelters are encouraged to prioritize services such as spay–neuter services or other needed veterinary procedures [9,12] to avoid unnecessary delays. For slow-track animals that are expected to have a longer average stay, shelters are advised to prioritize promotion and enrichment [1].

Limited shelter resources pose allocation questions. For example, cats in the top tier of cages may be seen by visitors more often than those in the bottom tier [13]. A subset of dog kennels may have higher visitor traffic and generate more adoptions [14]. These resources must be allocated to some subset of the animal population. An allocation that has the highest overall benefit is one that reduces average LOS and thus the in-care census count.

Our objective is to compare the effect of simple interventions on LOS and demonstrate whether they have more impact on fast-track (easier to adopt-out) or slow-track (harder to adopt-out) animals. Interventions are a limited resource. The shelter can send only a small number of animals to an adoption event, has a limited number of high-visibility kennels, and can only generate marketing videos for a subset of the animals. If these resources are targeted to achieve the most impact (reduction) on population average LOS, the shelter has fewer animals in care, provides better care with a fixed level of resources (such as staffing and kennels), and avoids exceeding its capacity.

The article is a mathematical study of the effect of resource allocation to slow-track and fast-track shelter animals. We consider only adoptions, because other outcome types entail additional metrics. Euthanasia is an undesirable outcome for which a metric such as the Live Release Rate would need to be considered alongside LOS. Return of found animals to their owners usually occurs quickly, and animals are available for adoption only after that path is deemed closed. Transfers to another organization delegate the adoption need and obscure the eventual outcome and LOS. To avoid these complications, the scope of this study is limited to adoptions—and the shelter subpopulation targeted for an adoption outcome.

## 2. Materials and Methods

Our approach entails three methods: analytical results examining an individual animal; steady-state population-level effects in a kennel allocation example; and a Monte Carlo simulation of the same example to account for the discrete stochastic intakes and adoptions. We begin with the definition of a baseline LOS distribution and then describe the details of these methods. Note that Section 2 includes the derivation of the analytical results, which are then plotted in Section 3.

### 2.1. Baseline LOS Distribution

We assume that the daily odds of adoption do not vary with days in care, making the LOS distribution geometric (except when interventions change the odds). With this memoryless distribution, our analysis applies to animals already in care as well as animals newly available for adoption.

In practice, distributions could have either increasing or decreasing adoption odds, depending on shelter circumstances. For example, without adequate enrichment activities, a dog may get stressed over time and become less adoptable. The opposite may also occur: a previously untrained dog that, in the shelter, learns to walk well on a leash becomes more adoptable. The constant odds model is a baseline in which the shelter maintains the initial adoptability of the animal.

The following positive quantities describe adoption and LOS. *D* is the daily odds of adoption and *P* the daily probability of adoption:

P(D)=D/(1+D)

*Q* is the daily probability of non-adoption:

Q(D)=1−P(D)=1/(1+D)

and *L* the expected length of stay:

L(D)=1/P(D)=(1+D)/D=1+1/D

These are all monotonic functions of each other. Fast-track animals have higher *D* and *P* and lower *Q* and *L*. Slow-track animals have lower *D* and *P* and higher *Q* and *L*.

We treat a day as a discrete unit to avoid the complications of intra-day events. Accordingly, we limit the baseline range of interest to *L* ≥ 2 (*D* ≤ 1).

We treat interventions as having a multiplicative effect on *D*. This means that an intervention changes adoption odds by the same factor on any animal to which it is applied. This analysis, then, is not applicable to interventions that impact animals in different ways (e.g., an adoption event targeting specific categories of dogs).

### 2.2. The Effect of One-Day Marketing

We want to determine the effect of one day of marketing, dependent on the pre-marketing baseline LOS. While the intervention may last for multiple days, we assume that the decision to continue can be made daily, and we model the one-day impact. Examples of marketing are placing the animal in a showcase area or featuring the animal online or in special events. The single day of marketing changes the odds by a factor *A* > 1 (the inequality signifying that the marketing intervention is raising adoption odds).

Let *L_X_* be the expected LOS if the animal has one day of marketing followed by return to the baseline condition. *L_X_* is a function of *A* and *D*:

$$L_{X}(A,D)=P(AD)+(1+L(D))Q(AD)$$

Let *X* be the impact of marketing on LOS, i.e., the difference in expected LOS between *L_X_* and the baseline. Then,

$$X(A,D)=L_{X}(A,D)-L(D)=1-P(AD)L(D)=(1-A)/(1+AD)$$

The intervention reduces expected LOS, i.e., *X* < 0. We take the partial derivative of *X* with respect to *D*:

$$\partial X(A,D)/\partial D=A(A-1)/{\left(1+AD\right)}^{2}>0$$

Therefore, reduction in LOS induced by one day of marketing is a monotonically increasing function of *D*. For any given A, *X* decreases monotonically with baseline *L*. Recalling that *X* < 0, |*X*| increases with *L*.

### 2.3. The Effect of Continuous Marketing

If we look at marketing interventions that continue until the animal is adopted, such as the permanent assignment of a higher-visibility kennel, the change in LOS is simply *L*(*AD*) − *L*(*D*). But since the resource must be used for an average of *L*(*AD*) days, we normalize by *L*(*AD*). This LOS change per day of use of the limited resource is, in fact, the same as the *X*(*A*,*D*) defined above.

X(A,D)=1−L(D)/L(AD)=(1−A)/(1+AD)

Thus, the reduction in LOS induced by continuous marketing until adoption, normalized by the number of days of usage, is monotonic in baseline L.

### 2.4. Marketing Effect on Continuous Distribution

We briefly examine LOS as a continuous variable following an exponential distribution, i.e., constant hazard. Let marketing alter the rate parameter *λ* of an exponential distribution multiplicatively to *λA*. Analogously to *X*(*A*,*D*) derived in Section 2.3, let *Y*(*A*, *λ*) be the LOS change normalized by the number of days of marketing:

Y(A,λ)=1−(1/λ)/(1/λA)=1−A

This impact is independent of *λ*, i.e., the intervention has a fixed benefit regardless of the baseline LOS. If the LOS is heavy-tailed, a continuous Weibull distribution with shape parameter 0 < *k* < 1 is more appropriate than exponential distribution. In the Weibull case, if the marketing effect is proportional on the scale parameter but leaves the shape parameter unchanged, the above result still applies.

### 2.5. Marketing Effect for Constant Hazards Within Each Intra-Day Adoption Window

One alternative to the proportionality of odds articulated in Section 2.2 is a type of punctuated exponential distribution. Starting from a geometric (discrete) view, we use the exponential distribution (constant hazard) to model the impact of marketing interventions during each day’s adoption window of fixed duration *T*. Since the rate *λ* rises to *λA* with marketing, the corresponding daily odds change from a baseline

$$D=e^{\lambda T}-1$$

To

$$D_{A}=e^{\lambda TA}-1={\left(1+D\right)}^{A}-1$$

Let *Z*(*A*, *D*) be the LOS change normalized by the number of days of marketing, analogous to *Y* above and *X* in Section 2.3:

$$Z(A,D)=(1+1/D)/{\left(1+D\right)}^{A}-1/D={\left((D+1\right)}^{1-A}-1)/D$$

If *D* ≪ 1, we can substitute 1 + *AD* for (1 + *D*)*^A^* and this becomes equal to *X*(*A*,*D*).

### 2.6. Illustrative Steady-State Allocation Example

We constructed an example (not modeling an actual shelter) with assignment of dogs to different kennel types as the marketing mechanism. The shelter’s standard kennels are the reference for baseline LOS, via which animals are divided into slow- and fast-track. For slow-track animals, we assigned baseline *L* = 27 (corresponding to daily odds 0.0385 and adoption probability 0.0370). For fast-track animals, we assigned baseline *L* = 3 (daily odds 0.500 and probability 0.333). We set daily intake to two animals per day, one for each track.

The shelter has 18 standard kennels, 7 showcase kennels with an adoption odds multiplier *A* = 1.5, one spotlight kennel with *A* = 3, and overflow kennels which are non-viewable with *A* = 0.5. These values of *A* are based on effects observed in dog adoptions: kennel viewing boosts adoption rates by a factor of 1.5 [15]; a small subset of viewable dogs experiences 3.8 times more adoptions than expected [16]. Proportional daily odds from Section 2.2 and Section 2.3 were used here, rather than the more complex effect of Section 2.5.

We tested two kennel assignment modes. In S priority, slow-track animals are preferentially assigned to the spotlight and showcase kennels (and then standard and overflow as needed). In F priority, fast-track animals get this preferential assignment.

For each combination of kennel type and animal track, we computed the steady-state in-care census count. We also computed the corresponding flow (adoptions per day) from the relationship LOS = in-care census count/flow.

### 2.7. Monte Carlo Simulation

To examine whether the discrete, stochastic nature of intakes and adoptions alters the result of the above steady-state calculation, we carried out Monte Carlo simulations of the S and F priority modes.

A simulation day is treated as a unit. We did not model intra-day time of arrival or departure. Starting from an occupancy state, we sample adoption outcomes from a binomial distribution based on the *P*(*AD*) for each combination of kennel and track. We optionally allow upgrades to fill vacancies in higher-value kennels. We then sample intakes from a Poisson distribution and assign them to kennels. We apply the priority being tested (S or F) throughout this process.

To generate near-independent samples, we started each Monte Carlo sample at the (discretized) steady-state values, simulated for 142 days (6 times the LOS of a slow-track dog in a standard kennel) to obliterate the initial state, and recorded the resulting in-care census as one independent sample. We produced 90,000 such samples, sufficient to bring the standard error to 0.02 for the in-care census total (and smaller by kennel type or track). Thus, the use of standard error is unnecessary in comparisons, and the results are subject only to the assumptions inherent in the structure of the model. We used Python 3.11.13, the pandas module 2.3.2, and Spyder IDE 6.0.8 on MacOS 26.3.

## 3. Results

We first report the analytical results, then examine the population-level marketing effects in the steady-state example and the Monte Carlo simulation.

### 3.1. Marketing Impact

**Figure 1:**
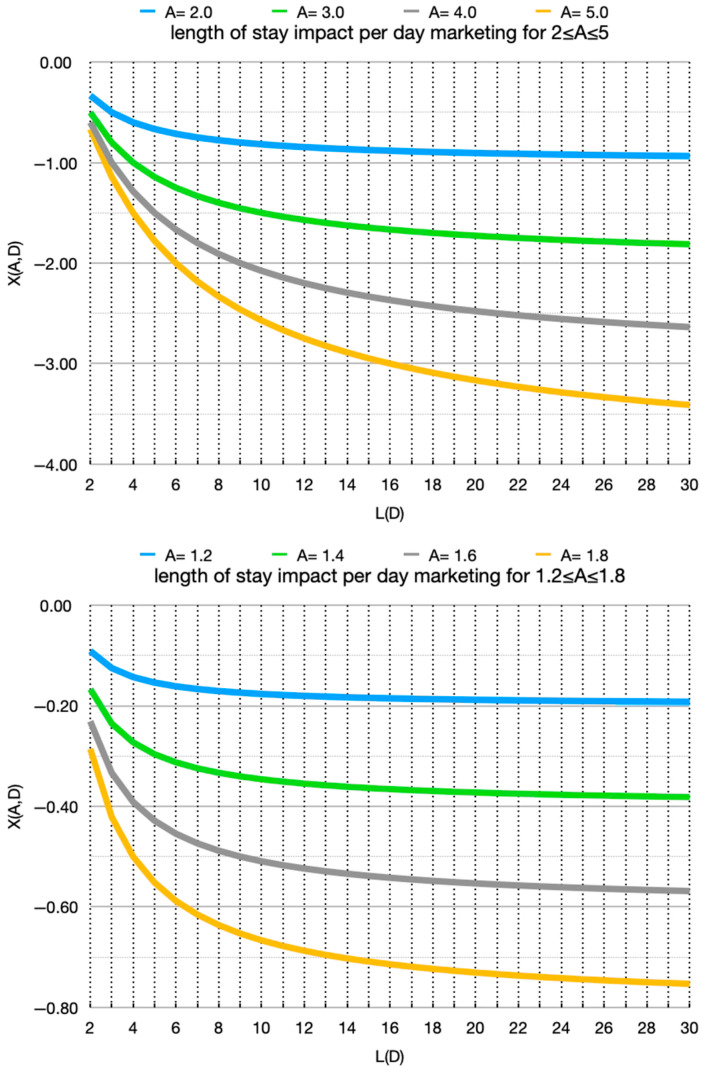
Expected change in LOS induced by marketing. Without marketing, the daily adoption odds are *D* and the corresponding baseline LOS is *L*(*D*). With marketing, the adoption odds change by a fixed factor *A*. The change in LOS induced by the marketing is *X*(*A*,*D*). For interventions limited to one day, the animal, if it is not adopted, returns to the baseline adoption odds. For interventions until adoption, the impact is normalized by the expected days of use. The top figure shows interventions with a large impact; 2 ≤ *A* ≤ 5. The bottom figure shows more modest interventions; 1.2 ≤ *A* ≤ 1.8.

For *X*, the LOS impact of one-day marketing or the per-day impact of ongoing marketing till adoption, we showed that *X* < 0 (LOS is lowered) and |*X*| monotonically increases with L. We visualize this (Figure 1) separately for 2 ≤ *A* ≤ 5, representing interventions with large impact on adoption odds, and for 1.2 ≤ *A* ≤ 1.8, representing a modest boost to adoption odds.

Slow-track animals (higher baseline LOS) have larger reductions in LOS. Therefore, marketing directed to them will therefore result in larger reductions in population average LOS and in the total in-care census count. For example, for *A* = 1.6, the LOS reduction is 0.23 per day at *L* = 2 and 0.57 at *L* = 30. The difference is a reduction of the in-care census count by 0.34 if the preferred kennel is allocated to the slow- rather than fast-track animals. For *A* = 4.0, the corresponding improvement is from 0.60 to 2.64, i.e., preferring the slower-track animal for the one-day marketing intervention saves 2.04 days of stay. If available daily, such an intervention translates to an ongoing reduction of the in-care census count by 2.04. At a large baseline *L*(*D*), hence *D* approaching 0, *X*(*A*,*D*) approaches 1 − *A*. For the curves in Figure 1, the asymptotic value for each curve is the horizontal gridline immediately below it. The gap between the curve and that gridline is the maximum remaining possible impact for *L*(*D*) > 30, and it is much smaller than the impacts within each curve, up to *L*(*D*) ≤ 30. The gains are thus primarily concentrated in low and medium baseline LOS values.

The exponential or Weibull distributions (with the marketing intervention having a proportional effect on the rate or scale parameter) yield impact that is indifferent to the baseline LOS. However, discrete distributions are more appropriate, because adoptions take place for only a modest fraction of the day, while census matters primarily overnight.

**Figure 2:**
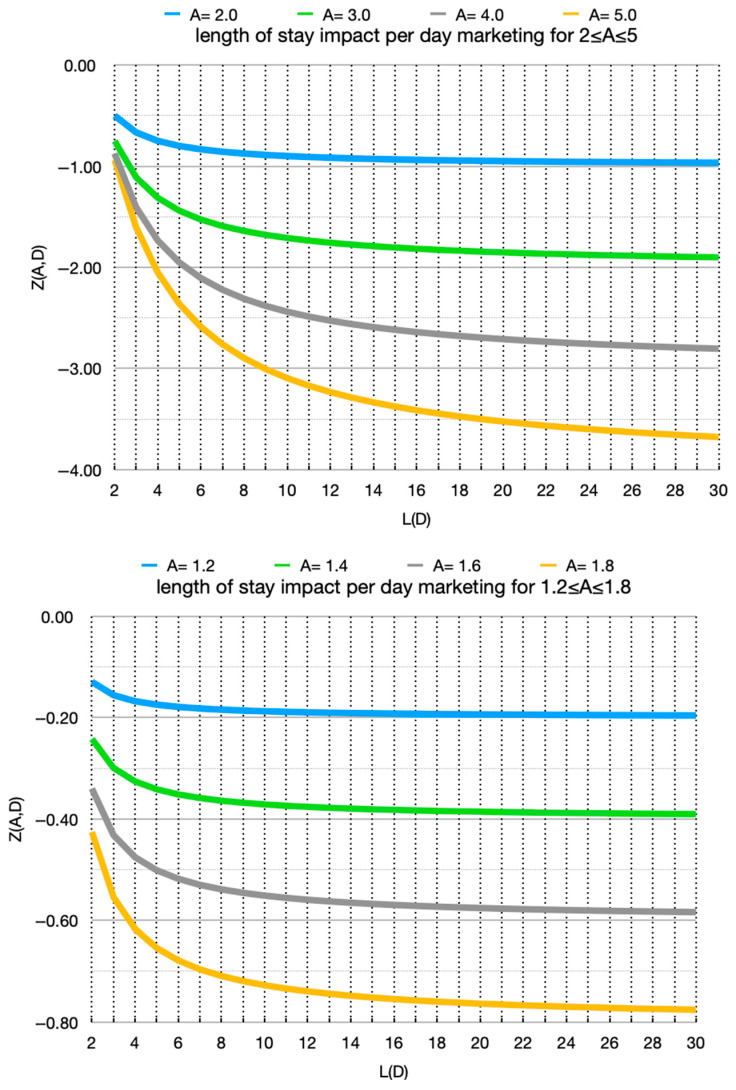
Expected change in LOS induced by marketing with constant hazard within intra-day adoption windows. Without marketing, the daily adoption odds are *D* and the corresponding baseline LOS is *L*(*D*). With marketing, the effect on adoption odds is computed for an adoption window as described in Section 2.5. *Z*(*A*,*D*) is the change in LOS normalized by the expected days of use.

Figure 2 shows the corresponding curves under the alternative formulation in Section 2.5, in which the marketing effect on adoption odds is non-proportional, relying instead on an exponential distribution within each day’s adoption window. The results are qualitatively similar to those of Figure 1. Thus, a continuous view of each day’s adoption window produces similar results as the simpler, discrete view. In the limit of large baseline *L*(*D*), *Z*(*A*,*D*) approaches 1 − *A*, the same limit as *X*(*A*,*D*). This is, for each curve, the horizontal gridline immediately below it.

### 3.2. Illustrative Steady-State Kennel Allocation Example

Our constructed shelter example has 18 standard kennels (*A* = 1), 7 showcase kennels (*A* = 1.5), one spotlight kennel (*A* = 3), and as a last resort overflow kennels (*A* = 0.5). In standard kennels, slow-track animals have an LOS of 27 and fast-track animals an LOS of 3. Each track averages one intake per day.

In a steady-state analysis, the total in-care census count (Table 1) is lower by 1.23 (5%) with S priority (slow-track animals prioritized for the preferred kennels) over F priority (fast-track animals prioritized). This is driven by a 1.1 (6%) reduction of animals in standard kennels. The census in overflow kennels is 0 under S priority but 0.13 under F priority.

Looking at the flows (daily adoptions), the spotlight kennel achieves only one adoption every 10 days under S priority, but more than one every 2 days under F priority, a five-fold advantage. For showcase kennels there is two-fold advantage under F priority. This is a consequence of assigning fast-track animals to these kennels. The price, however, is that standard kennels end up with more slow-track animals and have 2.3-fold fewer adoptions under F priority.

These flows may draw a shelter’s attention away from average LOS, leading to misleading conclusions. Spotlight and showcase kennels are a precious resource, and shelters may consider more adoptions occurring out of these kennels as an indicator of good utilization. But maximizing flow through this subset of kennels is directly tied to lower flow through the standard kennels. At the population level, this results in longer stays and higher resident census. The shelter should consider the global effect on its whole facility and in-care census, rather than any local metric for a subset of kennels or other marketing resources.

### 3.3. Monte Carlo Example

The purpose of the simulation is to show whether a discrete, stochastic analysis diverges from the steady-state conclusions. Standard errors will not be given, because sufficient simulations were carried out to render them irrelevant.

If upgrades (movements of animals already in care) are allowed, the in-care census count gap between S and F priorities diminishes modestly to 1.02 (4%) (Table 2). The total census count is higher than the steady-state census count, by about 1 in each strategy. The random fluctuations leading to occasional use of overflow kennels with lower adoption odds are the root cause of the higher total. Both priorities resort to overflow kennels—in which S priority has a lower census count by 0.5 (15%).

Without upgrades (i.e., vacant kennels await new intakes), there is an increase in in-care census totals by 1.49 under S priority and 1.90 under F priority, i.e., the advantage of S over F priority increases to 1.43 (Table 2). Note that now the spotlight and showcase kennels are slightly below capacity, since they remain sporadically vacant until new intakes are assigned to them.

Overall, the Monte Carlo simulation agrees with the simpler steady-state analysis. The advantage of S priority is slightly elevated with upgrades but diminished without upgrades. The simulation reveals some variance-driven overflow (superimposed on mean behavior) which may have operational consequences.

## 4. Discussion

Like complex organizational endeavors in other sectors of the economy, animal sheltering involves facets and subsystems which must be interconnected to achieve overall objectives. Unlike in other disciplines, animal sheltering research seldom entails industrial and systems engineering perspectives. In other sectors, there is a great deal of theoretical research on process design, modeling, monitoring, and control using models of the underlying processes rather than the details of specific instances. A study may address one element of the process hierarchy, rather than the full span, and still contribute to advancing process efficiency or reliability. Analogously, animal sheltering systems research need not always rely on experimentation at either the animal level or the shelter level. (In the United States, rigorous research studies with active participation by animal shelters are not common. Aside from ethical and logistical impediments, for government-operated shelters the needed approvals extend beyond shelter management to other offices in the local government apparatus.) A study may consider only one link in the chain of interactions that determines the efficacy of animal shelter operations. Theoretical and mathematical studies are a complement to, not a replacement for, other types of ongoing research.

This paper addresses a narrow component of a larger emerging framework of population-level modeling and resource allocation [5,6,7,8]. Using a simple model, we formalize the recommendation of prioritizing slow-track animals for marketing interventions as a more efficient path to reducing a shelter’s daily census count and mean LOS. As research identifies and resolves more of these narrow problems, global models and methodologies can advance to more effective optimization across heterogeneous populations.

While the recommendation already exists, how much it is actually followed is unknown. Were it standard practice, it would not need to be promoted in pamphlets and seminars. No model-based justification of the recommendation was articulated prior to this study. Consequently, no information existed on the magnitude of the effect or the range of LOS where it matters the most, and this study fills a gap. Using a discrete view of LOS, the framework of this paper strengthens the credibility of the recommendation. Our example simulations assume multiple levels of kennel visibility which exist only in some shelters, but these are stand-ins for other types of adoption promotion that are broadly available. The simulations show a potential census reduction of 4–5% when slow-track animals are preferred over fast-track ones.

We show the size of the impact—by baseline LOS and odds multiplier. We thus show at what LOS ranges the impact is operationally significant. The convexity of the curves in Figure 1 and Figure 2 means that the effect is much smaller in comparing a (baseline) LOS of 13 to 23 than 3 to 13. The effect is front-loaded at the shorter baseline LOS. Recalling that at high *L*(*D*) the limit for the impact is 1 − *A*, very little opportunity remains once baseline LOS exceeds 30. The study also shows what odds multipliers, i.e., *A* values (marketing impact), have operationally significant impact. This tells a shelter what resolution matters in the estimation of baseline LOS. Resolving low vs. medium baseline LOS helps, but medium vs. high less so. This may seem counterintuitive, but it occurs because we treat the intervention as a daily limited resource. We are computing the gain not per animal but rather per day of use of the resource. This is the appropriate view for, say, a more visited kennel or a daily social-media feature.

The study models only adoption outcomes and the animals that are eligible for adoption, because our focus is the allocation of limited marketing resources between slow- and fast-track animals, not the tradeoffs and competition among different outcome types.

This study uses a Monte Carlo simulation on animal shelter census and adoptions. Though simple, the simulation is useful in two ways. It shows that the discrete-unit nature of intakes and outcomes does not remove the advantage of preferential marketing of slow-track animals. It also shows that the randomized intakes and outcomes lead to sporadic overflow, even when steady-state calculations show sufficient capacity.

The LOS gains are not dramatic, but they are operationally meaningful. Importantly, they do not involve additional expenditure but rather optimal allocation of existing resources. Advice that aligns with the findings of this study is already available to shelters [1], but quantitative models of the type presented can further promote good practices.

It is tempting for a shelter to allocate limited marketing resources based on maximizing the probability that the intervention will result in an adoption, e.g., by sending fast-track animals to off-site adoption centers or events [14]. But marketing should not be judged by the prospect of a quick result. Its real value is medium- and long-term reduction in the census of animals in care, and this can be best achieved by marketing slow-track animals. In fact, highly adoptable animals may attract more visitors and adopters [14] during their stay, benefitting the in-care population as a whole. Many proactive shelters use checklists to apply marketing tools (kennel choice, events, bios, videos, social media) on long residents [14]. These are de facto slow-track, but so are newly arrived animals whose characteristics predispose them to slower adoption. Optimal allocation can help a shelter stay within its capacity, avoid the use of overflow kennels, and provide better care for all animals.

The constant-odds assumption is used here to obtain closed-form results. The framework can be extended to time-varying odds, but this would be an additional obstacle for shelters. Shelters refer to the qualitative contrast between slow- and fast-track animals, rather than a full estimate of each animal’s baseline expected LOS, because detailed LOS analysis is not in use. Even the quantification of population-wide changes in LOS by calendar period, as currently used, has been shown to be faulty [17]. This study is a simple but logical step forward.

If we treat LOS as a continuous variable and retain the assumption that the intervention has a proportional effect on the rate parameter; the impact of the intervention is independent of the baseline LOS. Thus, the guideline for preferring slow-track animals is not valid in this scenario. This applies to an exponential distribution as well as to a Weibull distribution which, with shape parameter 0 < *k* < 1, has heavier tails than the exponential. The discrete form of LOS is thus an essential component of the marketing preference guideline.

This study does not rank interventions, only their allocation *to the animals in care.* We use kennel categories as a canonical marketing intervention, because our assumption of the proportionality of the odds corresponds to a change in adopter traffic or attention. Any other action which can be reasonably assumed to have a multiplicative effect on the odds has the same properties. For example, general-purpose social-media promotion, generic adoption events, or enhanced website content. On the other hand, some interventions are inherently more powerful for a specific subset of adoptable animals. For example, special adoption fairs for senior or disabled animals, or social-media videos featuring skills that only some animals have. In the opposite direction, a high-traffic kennel may be too stressful for some animals. If the orientation of marketing kennels makes them substantially hotter or colder than other kennels, they may be seasonally inappropriate for dogs that do not tolerate the higher or lower temperatures. Shelter staff routinely make such animal-level refinements. A less common skill is an understanding (mathematical or intuitive) of stochastic processes, optimization, and the bearing these have on LOS and census.

The practical side of our study can be summarized as follows. First, check that the opportunity is time-bound, i.e., gives an adoption boost precisely for the days on which it is applied, because that is the only case we have analyzed here. In choosing a resident animal to prioritize, select among animals that are suited to the opportunity and are expected to have longer remaining stays. Avoid animals with short expected remaining stays (less than 10 days) when other suitable candidates exist. Among animals with expected remaining stays exceeding 20 days, the difference is too small to matter, and the choice can be a judgment call on individual animal characteristics. There is synergy, not competition, between staff judgment and mathematical guidelines. Shelters can judge when animals should be preferred or excluded, or if marketing should include a changing mix of animals to appeal to a broad adopter pool, all while keeping in mind the advantages of the optimal targeting of interventions to minimize average LOS and total in-care census count. Future work may produce mathematical and computational resources that can use a shelter’s data to generate shelter-specific insights.

## 5. Conclusions

This study provides a quantitative basis for the recommended practice of prioritizing slow-track animals for marketing interventions in animal shelters. Using a geometric LOS distribution and a multiplicative model of marketing impact on adoption odds, we showed analytically that the per-day LOS reduction from a marketing intervention is a monotonically increasing function of baseline LOS. Consequently, allocating limited marketing resources (such as high-visibility kennels or online promotions) to slow-track animals yields greater reductions in mean LOS and in-care census than allocating the same resources to fast-track animals.

A steady-state kennel allocation example illustrated a census reduction of approximately 5% under slow-track priority compared to fast-track priority, and Monte Carlo simulations confirmed this advantage for stochastic intakes and adoptions. Notably, the benefit is most pronounced at low-to-moderate baseline LOS values. The marginal gain diminishes at baseline LOS values approaching or exceeding 30 days. The discrete nature of daily LOS is essential to this result. Under continuous exponential or Weibull distributions with proportional rate effects, the intervention benefit is independent of baseline LOS, and the slow-track preference does not follow.

These findings do not require additional resources—only a reallocation of existing ones. They complement staff judgment that may tailor interventions to the needs of individual animals. The findings are consistent with operational guidance already available to shelters, but they offer quantification and refinement not previously present in the guidelines. Future work may extend the framework to time-varying adoption odds, competing outcome types, and shelter-specific data to support more refined resource allocation decisions.

## Figures and Tables

**Table 1:** In-care census and flows under steady-state analysis.

	Spotlight	Showcase	Standard	Overflow	Total
**In-care census: S priority**					
Slow	1.00	7.00	13.90	0.00	21.90
Fast	0.00	0.00	3.00	0.00	3.00
**Total**	**1.00**	**7.00**	**16.90**	**0.00**	**24.90**
**In-care census: F priority**					
Slow	0.00	6.07	18.00	0.13	24.20
Fast	1.00	0.93	0.00	0.00	1.93
**Total**	**1.00**	**7.00**	**18.00**	**0.13**	**26.13**
**Flow: S priority**					
Slow	0.103	0.382	0.515	0.000	1.000
Fast	0.000	0.000	1.000	0.000	1.000
**Total**	**0.103**	**0.382**	**1.515**	**0.000**	**2.000**
**Flow: F priority**					
Slow	0.000	0.331	0.667	0.002	1.000
Fast	0.600	0.400	0.000	0.000	1.000
**Total**	**0.600**	**0.731**	**0.667**	**0.002**	**2.000**

Under S priority, slow-track animals are preferentially housed in the kennels with higher adoption odds (left-to-right order). Under F priority, fast-track animals receive this preference. Flow is the daily number of adoptions. Total rows (in bold) show in-care census and flow summed across tracks for each kennel type and (in the last column) the total for the whole shelter.

**Table 2:** Monte Carlo simulation of in-care census.

	Spotlight	Showcase	Standard	Overflow	Total
**With upgrades: S priority**					
Slow	1.00	7.00	13.37	1.05	22.42
Fast	0.00	0.00	2.04	1.59	3.64
**Total**	**1.00**	**7.00**	**15.41**	**2.64**	**26.06**
**With upgrades: F priority**					
Slow	0.48	6.08	15.22	2.87	24.65
Fast	0.52	0.92	0.74	0.24	2.43
**Total**	**1.00**	**7.00**	**15.96**	**3.12**	**27.08**
**No upgrades: S priority**					
Slow	0.92	6.31	12.87	4.26	24.36
Fast	0.06	0.46	1.74	0.93	3.19
**Total**	**0.98**	**6.77**	**14.61**	**5.19**	**27.55**
**No upgrades: F priority**					
Slow	0.66	5.56	13.46	6.74	26.41
Fast	0.30	1.02	1.00	0.24	2.57
**Total**	**0.96**	**6.58**	**14.46**	**6.98**	**28.98**

Under S priority, slow-track animals are preferentially housed in the kennels with higher adoption odds (left-to-right order). Under F priority, fast-track animals receive the preference. With upgrades, movement of animals already in care is used to fill superior vacant kennels. Without upgrades, vacant kennels await new intakes. Total rows (in bold) show in-care census summed across tracks for each kennel type and (in the last column) the total for the whole shelter.

## Data Availability

The code used for the steady-state and Monte Carlo results is available in a repository [18].
